# 异基因造血干细胞移植后HHV-6病毒感染的临床特征分析

**DOI:** 10.3760/cma.j.cn121090-20240831-00330

**Published:** 2024-11

**Authors:** 莉 黄, 婷婷 韩, 芳芳 魏, 晓甦 赵, 于谦 孙, 晓冬 莫, 萌 吕, 翼飞 程, 兰平 许, 晓辉 张, 晓军 黄, 昱 王

**Affiliations:** 1 北京大学人民医院、北京大学血液病研究所、国家血液系统疾病临床医学研究中心，造血干细胞移植治疗血液病北京市重点实验室，北京 100044 Peking University People's Hospital, Peking University Institute of Hematology, National Clinical Research Center for Hematologic Disease, Beijing Key Laboratory of Hematopoietic Stem Cell Transplantation, Beijing 100044, China; 2 海南省人民医院/海南医科大学附属海南医院，海口 570311 Hainan General Hospital, Hainan Affiliated Hospital of Hainan Medical University, Haikou 570311, China

**Keywords:** 异基因造血干细胞移植, HHV-6病毒, 移植物抗宿主病, 血小板延迟植入, Allogeneic hematopoietic stem cell transplantation, Human herpes virus 6, Graft versus host disease, Delayed platelet engraftment

## Abstract

**目的:**

分析异基因造血干细胞移植（allo-HSCT）后100 d内人类疱疹病毒6型（HHV-6）激活的临床表现，研究HHV-6病毒载量对临床表现的影响及抗病毒治疗对HHV-6激活转归的影响。

**方法:**

回顾性研究2016年2月至2023年2月在北京大学血液病研究所接受allo-HSCT后100 d内HHV-6阳性的患者，分析患者的年龄、移植类型等基本特征以及临床表现，同时对移植后合并症等情况进行分析。

**结果:**

研究纳入305例HHV-6感染患者，HHV-6检测阳性中位时间为移植后20 d。有15例患者无症状，其余患者根据临床表现发生率由高到低依次是：发热、皮疹、腹泻、出血性膀胱炎、血小板延迟植入、中枢神经系统症状、腹痛、肺炎和口周麻木。189例患者合并急性GVHD（其中45例HHV-6感染发生在急性GVHD之前，120例HHV-6感染发生在急性GVHD之后）。45例患者有HHV-6定量检测结果，病毒滴度高低在临床表现中差异无统计学意义。108例（35.41％）患者存在与其他病毒共激活情况，合并感染的病毒包括巨细胞病毒、BK病毒、EB病毒等，其中合并EB病毒激活是影响总生存（OS）的独立危险因素。抗病毒治疗与未抗病毒治疗组患者HHV-6转阴时间差异无统计学意义［7（5～10）d对8（4～14）d，*P*＝0.199］，5年OS率差异也无统计学意义［（82.7±2.6）％对（91.3±3.1）％，*χ*^2^＝3.304，*P*＝0.069］。

**结论:**

allo-HSCT后HHV-6感染患者临床表现以发热、皮疹和腹泻多见，与病毒滴度高低无明显相关性，且是否抗病毒治疗在HHV-6转阴时间与移植后5年OS率中未见显著性差异。

## Abstract (en)

**Objective:**

This study aimed to analyze the clinical manifestations of human herpesvirus 6（HHV-6）infection within 100 days after allogeneic hematopoietic stem cell transplantation（allo-HSCT）and to investigate the association of HHV-6 viral load with clinical outcomes as well as the effect of antiviral treatment on the course of HHV-6 infection.

**Methods:**

This retrospective study included patients who tested positive for HHV-6 within 100 days after allo-HSCT at the Peking University Institute of Hematology from February 2016 to February 2023. The study analyzed the patients' baseline characteristics, including age and transplantation type, as well as their clinical manifestations. Additionally, post-transplant complications were examined.

**Results:**

This study detected that 305 patients with HHV-6 infection were positive with a median time of 20 days post-transplant. Fifteen patients were asymptomatic, whereas the remaining patients exhibited the following symptoms: fever, rash, diarrhea, hemorrhagic cystitis, delayed platelet engraftment, central nervous system symptoms, abdominal pain, pneumonia and perioral numbness. Acute graft-versus-host disease（aGVHD）was diagnosed in 189 patients, with 45 cases of HHV-6 infection occurring before the onset of aGVHD and 120 cases occurring after aGVHD developed. Quantitative HHV-6 detection was available for 45 patients, and no statistically significant differences were found in the clinical manifestations according to the viral titer. A total of 108（35.41％）patients experienced coactivation with other viruses, including cytomegalovirus, BK virus, and Epstein-Barr virus（EBV）. Notably, coinfection with EBV was determined as an independent risk factor for overall survival（OS）. No statistically significant difference in the time to HHV-6 viral clearance was observed between the antiviral treatment and non-treatment groups［7（5–10）days *vs* 8（4–14）days, *P*＝0.199］. Similarly, the 5-year OS rates between the two groups were not significantly different［（82.7 ± 2.6）％ *vs*（91.3 ± 3.1）％, *χ*²＝3.304, *P*＝0.069］.

**Discussion:**

The most prevalent clinical manifestations were fever, rash, and diarrhea in patients with HHV-6 infection after allo-HSCT. No significant correlation was found between the severity of the clinical symptoms and the viral titer. Additionally, no significant differences in the time to HHV-6 clearance or 5-year OS were observed between patients who received antiviral treatment and those who did not.

异基因造血干细胞移植（allo-HSCT）是目前治疗一些血液系统恶性肿瘤和非恶性血液疾病的唯一根治性手段。但是allo-HSCT过程中的预处理方案及移植后的免疫抑制治疗增加了移植后病毒感染或再激活的机会，而病毒感染是移植后患者非复发死亡的主要原因之一。

人类疱疹病毒6型（HHV-6）属于人类疱疹病毒科，与其他人类疱疹病毒相似，HHV-6能够在宿主体内保持一种潜伏或持续状态，并在机体免疫低下的时候可能再激活[1]。不同中心报道allo-HSCT后HHV-6激活比例不等[2]–[3]。其中脐血移植患者HHV-6激活率高达75.0％～94.7％[4]–[5]。原发HHV-6感染多于2岁以内发病[6]，通过检测0～21月龄的幼儿的血清发现，0～5月龄儿童抗体阳性率从52％下降到5％，出生6个月后体内有抗体的儿童数量逐渐增加，HHV-6阳性率从6月龄的14％上升至12月龄的83％，几乎所有13月龄以上的儿童体内都有抗体。93％患儿有症状，主要表现为发热、烦躁、腹泻、皮疹和玫瑰疹等[7]。allo-HSCT后HHV-6感染的临床表现主要为发热、皮疹、中枢神经系统症状、肺间质病变等[2],[8]–[11]，但更详细的临床特点以及HHV-6病毒载量与临床表现、干预治疗的关系少有报道。我们中心既往研究了肠黏膜HHV-6检测在移植后重度腹泻患者中的意义[12]以及前瞻性HHV-6阴性及阳性病例临床特征的差异[13]。本研究旨在研究HHV-6激活后的具体临床表现，同时研究病毒载量对临床表现的影响，以及抗病毒治疗后HHV-6激活的转归情况，以期明确HHV-6感染在移植后的常见临床表现以及是否需要积极干预。

## 病例与方法

1. 病例：本研究为回顾性研究。纳入2016年2月至2023年2月在北京大学血液病研究所接受allo-HSCT且移植后100 d内HHV-6检测阳性的患者，收集患者年龄、移植类型、临床表现、移植后合并症等基本特征信息。

2. HHV-6检测方法：本研究所有样本均采用上海之江生物科技有限公司腺病毒核酸测定试剂盒（荧光PCR法）进行HHV-6病毒DNA检测，操作方法按照说明书。样本类型包括外周血、粪便、咽拭子、脑脊液、痰液、肺泡灌洗液（BALF）、尿液、组织。

3. 移植方案：同胞相合移植采用改良白消安（Bu）/环磷酰胺（Cy）预处理方案；单倍体移植及非血缘供者移植均采用改良Bu/Cy+抗胸腺细胞球蛋白（ATG）方案。移植物为rhG-CSF动员后的供者外周血干细胞±骨髓干细胞。采用环孢素A（CsA）、霉酚酸酯（MMF）及短程甲氨蝶呤（MTX）方案预防急性移植物抗宿主病（aGVHD）。以阿昔洛韦、复方磺胺甲噁唑及抗真菌药物预防病毒、耶氏肺孢子菌及真菌感染。对于巨细胞病毒（CMV）阳性的患者，予更昔洛韦（5 mg/kg）或者膦甲酸钠（60～120 mg/kg）抗病毒治疗。对于HHV-6阳性的患者，根据患者临床表现，部分给予更昔洛韦或膦甲酸钠抗病毒治疗。

4. 定义：中性粒细胞植活：移植后ANC>0.5×10^9^/L连续3 d；血小板植活：移植后在未输注血小板的情况下，PLT>20×10^9^/L连续7 d。aGVHD的诊断及分度参照《中国异基因造血干细胞移植治疗血液系统疾病专家共识（Ⅲ）急性移植物抗宿主病（2020年版）》[14]。HHV-6感染（HHV-6阳性）：应用PCR方法从外周血、活检组织或其他体液中检出HHV-6 DNA。血小板延迟植入（delayed platelet engraftment, DPE）定义为移植28 d后持续性PLT<20×10^9^/L。合并aGVHD：HHV-6感染前、后7 d内出现aGVHD。

5. 统计学处理：采用SPSS 26.0软件对数据进行统计分析。计量资料采用ShaPiro-Wilk进行正态性检验，呈正态分布用均数±标准差表示，组间比较采用独立样本*t*检验，呈非正态分布用中位数和四分位间距表示，组间比较采用Mann-Whitney *U*检验；计数资料用构成比（％）表示，组间比较采用卡方检验或Fisher确切概率法。采用Kaplan-Meier法绘制生存曲线，采用Cox等比例风险模型分析事件的影响因素。双侧*P*<0.05为差异有统计学意义。

## 结果

1. 病例资料：共305例患者allo-HSCT后100 d内HHV-6检测阳性，中位年龄20（1～58）岁。其中儿童126例，HHV-6检测阳性中位时间为移植后17（11～63）d；成人179例，HHV-6检测阳性中位时间为移植后20（7～98）d，成人HHV-6阳性时间较儿童滞后（*P*<0.05）。疾病类型：急性髓系白血病99例（32.46％）、急性淋巴细胞白血病115例（37.70％），再生障碍性贫血35例（11.48％）、骨髓增生异常综合征28例（9.18％）、其他28例（9.18％）。人类白细胞抗原（HLA）配型全相合9例（2.95％），HLA配型不全相合296例（97.05％）。回输单个核细胞（MNC）计数9.52（3.76～25.45）×10^8^/kg，CD34^+^细胞计数2.99（0.19～24.81）×10^6^/kg。粒细胞中位植入时间12（9～33）d，血小板中位植入时间14（7～76）d。

2. HHV-6感染患者的临床特征：

（1）症状分布情况：全部305例血HHV-6阳性患者中，完全无症状患者仅有15例（4.92％），其余患者均出现相关症状，262例（85.9％）患者合并发热，198例（64.92％）合并皮疹，100例（32.79％）合并腹泻，85例（27.87％）合并出血性膀胱炎，42例（13.77％）合并血小板延迟植入，22例（7.21％）合并中枢神经系统症状，17例（5.57％）合并腹痛，16例（5.25％）合并肺炎，1例患者合并口周麻木。

（2）同时检测的相关体液组织的HHV-6表达情况：合并腹泻的患者中有28例进行了粪便HHV-6检测，其中6例HHV-6阳性，22例HHV-6阴性；另外3例患者行肠镜取肠黏膜进行检测，其中1例HHV-6阳性，2例HHV-6阴性。1例合并口周麻木的患者，颊黏膜HHV-6阳性。22例合并中枢神经系统症状的患者中15例行腰椎穿刺进行脑脊液检查，7例HHV-6阳性，8例HHV-6阴性。

（3）合并aGVHD的情况：305例HHV-6感染患者中，189例（61.97％）患者合并aGVHD（45例HHV-6感染发生在aGVHD之前，120例HHV-6感染发生在aGVHD之后），其中Ⅰ度84例、Ⅱ度78例、Ⅲ度17例、Ⅳ度10例，Ⅱ～Ⅳ度aGVHD占55.56％。aGVHD部位：116例（61.38％）为单独皮肤型aGVHD，46例（24.34％）同时累及皮肤及肠道，15例（7.94％）单独累及肠道，8例（4.23％）同时累及皮肤、肠道和肝脏。

3. HHV-6病毒载量与临床表现：305例患者中45例接受HHV-6定量检测，HHV-6病毒载量中位数为26 600拷贝数/ml，故以26 600拷贝数/ml作为cut-off值，<26 600拷贝数/ml归为低滴度组，≥26 600拷贝数/ml归为高滴度组，结果如表1所示，在临床表现方面，低滴度组与高滴度组之间的差异无统计学意义。

**表1:** 异基因造血干细胞移植后HHV-6病毒高低滴度组临床特征比较

临床特征	低滴度组（22例）	高滴度组（23例）	统计量	*P*值
性别［例（％）］			5.020	0.025
男	15（68.2）	8（34.8）		
女	7（31.8）	15（65.2）		
年龄（岁，*x*±*s*）	33.77±15.39	36.96±14.07	−0.725	0.472
疾病类型［例（％）］			2.321	0.509
AML	10（45.5）	10（43.5）		
ALL	5（22.7）	7（30.4）		
MDS	2（9.1）	4（17.4）		
其他	5（22.7）	2（8.7）		
供受者性别是否相合［例（％）］			1.793	0.181
相合	13（59.1）	9（39.1）		
不合	9（40.9）	14（60.9）		
血型是否相合［例（％）］			0.184	0.668
相合	12（54.5）	14（60.9）		
不合	10（45.5）	9（39.1）		
单个核细胞计数［×10^8^/kg，*M*（范围）］	10.21（8.44～11.21）	9.52（7.40～11.17）	−1.113	0.266
CD34^+^细胞计数［×10^6^/kg，*M*（范围）］	2.81（2.05～5.18）	3.15（2.34～4.50）	−0.500	0.617
血小板是否延迟植入［例（％）］			1.889	0.169
否	5（22.7）	1（4.3）		
是	17（77.3）	22（95.7）		
血小板植入天数［d，*M*（范围）］	12.5（9～14）	13（10～17）	−0.719	0.472
是否发热［例（％）］			1.369	0.242
否	2（9.1）	5（21.7）		
是	20（90.9）	18（78.3）		
最高体温［°C，*M*（范围）］	38.6（37.7～39.3）	38.7（37.5～39.5）	−0.171	0.864
发热持续时间（d，*x*±*s*）	4.74±3.65	4.25±3.17	−0.207	0.836
皮疹［例（％）］	10（45.5）	13（5.5）	0.551	0.458
皮疹持续时间［d，*M*（范围）］	0（0～6）	3（0～6）	−0.375	0.707
腹泻［例（％）］	5（22.7）	6（26.1）	0.069	0.793
腹痛［例（％）］	0（0）	2（8.7）	Fisher	0.489
神经系统症状［例（％）］	1（4.5）	3（13.0）	0.228	0.633
脑脊液HHV-6病毒阳性［例（％）］	1（4.5）	2（8.7）	Fisher	1.000
肺部改变［例（％）］	1（4.5）	0（0）	Fisher	0.489
合并aGVHD［例（％）］	16（72.7）	19（82.6）	0.635	0.425
aGVHD部位［例（％）］			1.555	0.212
肠道	1（4.5）	0（0）		
肠道+肝脏	0（0）	1（4.3）		
皮肤	10（45.5）	10（43.5）		
皮肤+肠道	5（22.7）	6（26.1）		
皮肤+肠道+肝脏	0（0）	1（4.3）		
皮肤+肝脏	0（0）	1（4.3）		
aGVHD严重程度［例（％）］			2.905	0.088
0～Ⅰ度	16（72.7）	11（47.8）		
Ⅱ～Ⅳ度	6（27.3）	12（52.2）		
HHV-6转阴时间（d，*x*±*s*）	9.00±3.73	8.72±4.52	0.207	0.837

**注** 低滴度组：HHV-6病毒载量<26 600拷贝数/ml；高滴度组：HHV-6病毒载量≥26 600拷贝数/ml；AML：急性髓系白血病；ALL：急性淋巴细胞白血病；MDS：骨髓增生异常综合征；aGVHD：急性移植物抗宿主病

4. 合并其他病毒感染情况及转归：305例患者中，231例（75.74％）为单纯HHV-6感染，108例（35.41％）存在与其他病毒共激活情况，合并感染的病毒包括CMV、BKV、EB病毒（EBV）、HSV、JCV等（表2）。单纯HHV-6感染与HHV-6合并其他病毒阳性患者HHV-6转阴时间差异无统计学意义［7（5～11）d对7（5～11）d，*P*＝0.946］。其中连续随访患者296例，Cox回归分析结果显示合并EBV是影响总生存（OS）的独立危险因素（*HR*＝3.491，95％ *CI* 1.081～11.276，*P*＝0.037）。

**表2:** 异基因造血干细胞移植后100 d内HHV-6感染患者合并其他病毒共激活情况

共激活病毒	例数（％）
CMV	40（13.11）
BKV	49（16.07）
EBV	10（3.28）
HSV	9（2.95）
JCV	9（2.95）
IV	4（1.31）
细小病毒B19	3（0.98）
COVID-19	2（0.66）
RSV	1（0.33）
HBoV	1（0.33）
ADV	1（0.33）
鼻病毒	1（0.33）

**注** CMV：巨细胞病毒；BKV：BK病毒；EBV：Epstein Barr病毒，又称人类疱疹病毒4型；HSV：单纯疱疹病毒；JCV：JC病毒；IV：流感病毒；COVID-19：新型冠状病毒；RSV：呼吸道合胞病毒；HBoV：人类博卡病毒；ADV：腺病毒

5. 治疗与转归：抗病毒治疗组和未抗病毒治疗组患者HHV-6转阴时间分别为7（5～10）d和8（4～14）d，差异无统计学意义（*P*＝0.199）。305例HHV-6感染患者中，截至2024年9月13日，连续随访患者共296例，抗病毒治疗组和未抗病毒治疗组5年OS率分别为（82.7±2.6）％和（91.3±3.1）％，差异无统计学意义（*χ*^2^＝3.304，*P*＝0.069）。

## 讨论

allo-HSCT是治疗恶性血液病的重要手段，移植后HHV-6感染十分常见[15]。目前HHV-6感染主要通过检测外周血中HHV-6 DNA的存在来判断[16]。

HSCT后HHV-6感染的发生率较高，不同中心报道的HHV-6感染的发生率为22.0％～72.2％[8],[17]–[19]，HHV-6 DNA阳性多发生于移植后第2～6周[8],[20]–[22]，大多数情况下，阳性持续时间<3周[3],[8],[23]。本研究中305例HHV-6感染患者检测出阳性的中位时间为移植后20（7～98）d，其中成人HHV-6阳性时间较儿童滞后。

本研究中305例HHV-6感染的患者有15例（4.91％）无症状，其余患者根据临床表现发生率由高到低依次是：发热、皮疹、腹泻、出血性膀胱炎、血小板延迟植入、中枢神经系统症状、腹痛、肺炎和口周麻木。发热是HHV-6感染后最常见的临床表现，有262例（85.9％）患者发热，中位持续时间仅有6（1～31）d。198例（64.92％）患者出现皮疹，多数症状较轻，中位持续时间为6（1～40）d。皮疹是移植后aGVHD的主要表现之一，本研究中HHV-6感染患者中189例合并aGVHD（其中45例发生在aGVHD之前，120例发生在aGVHD之后），合并有皮肤表现的aGVHD患者170例，既往研究也证实HHV-6感染是移植后发生Ⅱ～Ⅳ度aGVHD的危险因素[13],[24]。虽然HHV-6与aGVHD之间的因果关系尚未得到证实，但某些数据支持这种关系的生物学合理性，例如有小规模的研究阐述了HHV-6复发与促炎性细胞因子反应（主要是IL-6浓度升高）有关[23]。同时，许多促炎性或Ⅰ型细胞因子（IL-5、IL-6、IL-10等）与aGVHD有关联[25]。本中心有45例患者随后发生aGVHD，但仍需进一步研究HHV-6对免疫系统的影响及了解HHV-6相关疾病的发病机制，另有120例HHV-6感染发生在aGVHD之后，考虑可能与抗GVHD治疗后免疫功能低下、病毒激活有关。本研究中腹泻患者100例（32.79％），与目前报道腹泻发生率基本一致[12],[26]，伴有腹痛的仅有5.57％。腹泻患者中28例进行了粪便HHV-6检测，其中阳性仅占21.43％，3例腹泻患者进行胃肠黏膜HHV-6检测，仅1例阳性。这与本中心既往一项研究结果一致：腹泻发生的时间、次数、量以及腹泻持续时间等方面在HHV-6阳性与阴性患者中差异均无统计学意义[12]。我们将完成定量检测的样本进行了统计，高滴度组与低滴度组之间aGVHD分度差异无统计学意义（*P*＝0.088），但本文中定量检测的样本量较少，如果扩大样本量，或许统计结果会更加有意义。这些患者HHV-6与aGVHD发生时间较近，二者互为因果，皮疹、腹泻等临床症状相似，对临床诊断以及鉴别诊断增加了一定的难度，因此治疗上仍需权衡患者移植模式以及具体情况综合判断，制定个体化治疗策略。延迟植入是病毒感染后很常见的合并症，本研究中仅1例患者中性粒细胞未植入，42例（13.77％）患者合并血小板延迟植入。Dulery等[27]研究结果显示，allo-HSCT后HHV-6感染与血小板植入延迟、移植后早期死亡率和aGVHD有关。而另有研究表明，在中性粒细胞和血小板恢复方面，HHV-6感染和未感染受者之间差异无统计学意义，但高水平的HHV-6 DNA与血小板恢复延迟相关[3]。目前已知HHV-6可感染造血祖细胞，减少体外集落形成，这可能解释了同种allo-HSCT后植入延迟的原因[28]–[29]。allo-HSCT患者在HHV-6感染后脑炎的发生率为1.4％～3％，且有研究表明高水平的血浆HHV-6 DNA可能与HHV-6脑炎发生的高风险相关[8],[30]。移植后发生HHV-6脑炎的患者临床预后较差[31]。本研究中22例（7.21％）患者合并中枢神经系统症状，15例进行脑脊液HHV-6检测，其中7例阳性，脑脊液阳性患者中仅1例进行定量，病毒载量高达1.19×10^6^拷贝数/L。提示我们如果在HSCT后出现中枢神经系统功能障碍伴高水平血浆HHV-6 DNA，则应警惕关注HHV-6脑炎风险。此外，合并肺炎的患者占5.25％，其中1例患者胸水HHV-6检测结果呈阳性。另有1例患者无诱因反复出现口周麻木，颊黏膜组织HHV-6检测阳性，送定量检测病毒载量为1.07×10^4^拷贝/L。提示浆膜腔积液及组织HHV-6检测对于临床诊断也可能具有重要的临床意义。

HHV-6病毒感染后所致临床表现是否和其滴度相关呢？既往研究显示高水平的血浆HHV-6 DNA可能与HHV-6脑炎发生的高风险相关[8],[30]，但也有研究显示，HHV-6感染血浆病毒载量与患者预后之间无明显相关性[9]。本研究中接受HHV-6定量检测的患者共45例，通过比较我们发现病毒滴度高低与临床表现无明显相关性，这很可能说明HHV-6阳性与是否出现临床症状不一定直接相关。因为本研究是回顾性研究，有15例（4.91％）患者无症状，若普遍进行HHV-6筛查，可能会发现更多无症状患者，这一结论与我中心既往前瞻性研究相符合[13]。

我们还进行了HHV-6感染合并其他病毒感染情况及治疗情况的生存分析。两种病毒同时感染可能的机制是由于移植后严重的免疫抑制所致，尤其在单倍体移植模式下[32]，另一方面可能的解释是，HHV-6感染后可通过抑制IL-12的产生来抑制机体的抗病毒免疫反应，进而促进HHV-6激活及其他病毒感染的发生[26],[33]–[34]。但是关于HHV-6病毒合并其他病毒感染是否对预后有影响的研究较少。本研究分析显示，是否合并其他病毒感染对HHV-6转阴时间差异无统计学意义，但对连续随访的296例患者进行生存分析显示，合并EBV是影响OS的独立危险因素。治疗转归方面，抗病毒治疗组和未抗病毒治疗组患者HHV-6转阴时间分别为7（5～10）d和8（4～14）d，差异无统计学意义，5年OS率差异亦无统计学意义。

综上所述，本研究数据以单倍体移植为主，在本中心的移植模式下，HHV-6感染是移植后常见的合并症，HHV-6感染患者临床表现以发热、皮疹和腹泻多见，这些表现与aGVHD难以鉴别。HHV-6感染临床表现与其滴度高低无明显相关性，且因HHV-6感染后无论治疗与否，HHV-6阳性持续时间均较短，5年OS率差异无统计学意义，这提示我们对移植后出现HHV-6感染，需要综合评估其意义，治疗决策需要根据临床情况作出。本研究为回顾性研究，具有一定的局限性。未来可能需要设计更合理的随机对照试验进一步了解移植后HHV-6感染的临床特征。
